# African bioethics: methodological doubts and insights

**DOI:** 10.1186/s12910-018-0338-6

**Published:** 2018-12-27

**Authors:** John Barugahare

**Affiliations:** Department of Philosophy, Makerere University, P. O. Box 7062, Kampala, Uganda

**Keywords:** African bioethics, Research ethics, Ethical imperialism, Critical thinking, Bioethical principles, Africanity, Ubuntu

## Abstract

**Background:**

A trend called ‘African bioethics’ is growing on the continent due to perceptions of existing bioethics, especially guidelines for international collaborative research, as ‘ethical imperialism’. As a potential alternative to ‘Western Principlism,’ ‘African bioethics’ is supposed to be indigenous to Africa and reflective of African identity. However, despite many positive insights in the on-going discussions, it is feared that the growth of bioethics in Africa lacks a clear direction. Some of the views threaten to distort the essence of bioethics and its development on the continent.

**Main text:**

This paper presents some of the dominant views on ‘African bioethics’, an examination of which reveals some valuable insights into the direction bioethics in Africa ought to take, but at the same time confirms some methodological challenges in some contributions to the discussion. On top of acknowledging critical insights in the discussion, the paper reveals that some views are characterized by arbitrariness and rhetorical discussions based on a strong negative and yet hard-to-accept assumption; doubtful designation; lack of a clearer problem being addressed and consequently obscure question(s) and aim(s) of the discourse. Finally, some methodological insights are proposed to guide bioethics research and scholarship in Africa. Specifically, the paper proposes that in search for the legitimacy of bioethics in Africa, we ought to protect the essence of bioethics by giving considerable attention to the utility of subsequent bioethics. To achieve this we need to specify the problem and proper designation for the discourse; focus on principles *qua* principles with impartiality and how to ensure their strict implementation; and encourage critical thinking as part of bioethics.

**Conclusion:**

In cultivating bioethics in Africa, the pursuit of identity is legitimate but must be conditional in light of other competing considerations. We should focus on an objective search for bioethical principles that can be effective in responding to African and global health challenges of moral significance, irrespective of the origin of the principles and at the same time focus more on strategies for ensuring compliance with resulting principles.

## Background

Whereas formal knowledge and practices of existing bioethics have their origin and deeper roots in the West, bioethics is still an emerging field of enquiry and practice in Africa. Some widely shared opinions that mark discussions of bioethics in Africa are claims that current bioethics is foreign to Africa and, therefore, lacks ‘Africanity’/‘Africanness’ or ‘authenticity’ in Africa [1, 2]; that an ‘African voice is not sufficiently included in the development of existing bioethics’ [3]; that the teaching and implementation of bioethics in Africa is an attempt by the West to re-colonize and dominate, and exploit Africa as one recent discussion reveals [4]. Consequently, a number of related works are characterized by concern about ‘ethical imperialism’ and consequently the decolonization of bioethics in Africa. For the good intentions of contributors to this discussion, one outstanding challenge facing the growth of bioethics in Africa is the looming distortion of its essence. Even though there are potentially many views on the essence of bioethics, given its background, its *raison d’etre* seems to be ensuring that the means by which we address our health needs and challenges are morally justifiable and socially acceptable, wherever such means are applied. The looming misrepresentation of the essence of bioethics seems to be a consequence of methodological difficulties that characterize some contributions to the discussion. These methodological problems are marked by disproportionate concern with assertions of ‘difference’, ‘identity’ ‘the indigenous’ and ‘tradition’ along with attempts to uncritically demonize their opposites such as ‘universality’, ‘otherness’, ‘the foreign’ and ‘modernity’. The threat of distortion becomes even stronger with the rhetoric of ‘Africanity’/‘Africanness’ and other terms with similar connotations.

Apart from a more recent discussion that has pointed at methodological flaws in African bioethics [4], concern was expressed earlier about the trend of bioethics in Africa when it was symbolically described as ‘sailing in a rough ocean using archaic technology’, to the extent that one is never sure where one is or what direction one ought to take [1]. In view of these methodological challenges, it has been observed that ‘As the decolonization process involves sound methodologies, there is need for robust methodological inquiry in bioethics in Africa [5]. The purpose of this paper is twofold: to highlight potential methodological pitfalls that characterize some contributions to bioethics in African and how they threaten to stifle its development and essence on the continent and to propose a basic method which can be developed further to guide the growth of bioethics in Africa. The discussion will present and review some scholarly literature on African bioethics and then suggest methodological problems that characterize these views. The paper ends with a proposal of methodological insights intended to guide the development of bioethics in Africa.

### Some dominant views on ‘African bioethics’

To date, some African bioethicists have correctly observed the lack of African voice in existing bioethics generally [2, 6]. Others have attempted to articulate specific bioethical principles from an African worldview [7], while at least some have gone further to propose a theory of African bioethics [8, 9]. At the same time, other contributors have attempted to confront the challenge of methodology or generally the rigour with which bioethics research and scholarship in African are proceeding; one, by emphasizing the importance of employing critical thinking in ‘African bioethics’ [1] and two, by explicitly calling for an inquiry into methodologies in African bioethics [4]. In these discussions generally, a widely implied foundational premise is that existing bioethical principles and guidelines are foreign to Africa. From this premise there follows a conclusion that existing bioethics lacks ‘authenticity’ in Africa [1]. In particular, current international bioethical principles and guidelines for health-related research, and their claim to universal validity, are widely criticized as both a colonial or neocolonial feature of the West intended to stifle the influence of other cultures in the development of global bioethics [6]. Consequently, there strong encouragement of *critical thinking* in discussions of bioethics in Africa. On this view critical thinking ‘would require *bioethicists from Africa to root their enquiries on ethical values in African traditions* … enable Africans to suspect or be suspicious of the moral authority and authenticity of *foreign principles and values*’ [1] (my emphasis). To preempt my later discussion, one plausible and unfortunate implication of these lines is, arguably, that without any further qualification, all ethical theories, principles, values, beliefs and consequent practices rooted in African traditions or indigenous to Africa, ought to always take precedence over those from the West whenever implemented in Africa.

Further, some contributors to the discussion bemoan ‘the lack of Africanness in African bioethics as Africanity is undergoing a process of erosion in the light of contemporary changes and faced with foreign values’ [1]. In the same spirit, bioethics in Africa seems to be primarily aimed at challenging what has been described as ‘a strong impulse to present Western bioethics and Western cultures as the only rational and universally valid ones’ [10]. Some non-African bioethicists share these sentiments. For example, some are concerned that existing international bioethics is ‘a discipline with some discernible biases, some unmistakable signs of *its heavily American origins*; [...]. Bioethics is often too American [...]’ [11]. Similarly, some have expressed discomfort with the fact that ‘medical science and technology, and the ethics designed to deal with their impact, are all Western in origin; [...]. Consequently, such values are often perceived as alien, and even antipathetic to many non-Western worldviews [12].

### Methodological problems

#### General outlook of views on African bioethics

One outstanding feature of discussions of bioethics in African is a decolonization imperative. This is not surprising given the range of appalling experiences associated with colonialism and apartheid described by some contributors to this discussion [1, 2, 13]. Whereas there is nothing inherently wrong with the decolonization of bioethics, the necessity of this decolonization will be stronger and urgent if, for example: 1) it is shown to be true that the promotion of existing bioethics in Africa is a neocolonial agenda that will *most likely* repeat the described evils or some that closely imitate them; 2) we can identify African-inspired theories and principles which are evidently superior to and incompatible with Western ones; and 3) we can demonstrate that western-based theories and principles are doing more harm than good in the management of contemporary African health systems. However, the problem that arises from concern with decolonization *qua* decolonization as it currently appears to be the case, is that it fosters arbitrary mistrust in current efforts to grow bioethics on the continent. This in turn negatively impacts our aptitude to think critically about the growth of bioethics in Africa. It limits our readiness for impartial criticism of our own ideas (as individuals or groups) and those of others; the exercise of rationality (making assertions basing on objectively verified evidence); and the like.

One example to quickly make intelligible the above contention: whereas it is true that there is need for critical thinking to question the moral authority and authenticity of foreign ethical principles and values [1], this habit of *self-criticism* does not appear to be equally encouraged in the process rooting our inquiry into African indigenous values. There is a commonly implied tendency to arbitrarily exempt traditional and indigenous values from similar critical scrutiny against the requirement of impartiality. Objectivity requires that inquiry into what will become African theories and principles of bioethics should not only be rooted in ethical values in African traditions but should employ similar skills in critically appraising our own traditions. This lack of impartiality is more evident in the following definition of ‘African bioethics’: ‘As such, by African bioethics, it should be understood as thinking that is rooted and flows from Africa’s innate traditional values’ [1]. On the converse, however, in my opinion, when we speak of bioethics in African we ought to mean something like, ‘thinking driven towards identifying all ethical principles, including those rooted in African values and those that may not be, which can effectively address contemporary Africa’s specific health needs and challenges’. Generally, an overbearing methodological error that characterizes some views on ‘African bioethics’ is a wide but uncritical sanctification of everything indigenous and traditional to Africa and an arbitrary censure of everything foreign and modern.

The above attitude (partiality) raises critical questions: What determines the desirability of a given ethical principle? Is it its place of origin or its author? Arguably, neither of these. Instead, for example, it could be a principle’s efficacy (ability to cause morally justifiable conduct) and the social acceptability of such a principle in a given society, or a combination of other factors (such as promotion of social harmony, cohesion, efficiency in resource use among others), but not the mere fact of such principle being indigenous or traditional to the concerned community. It is an error, therefore, for us to be disproportionately concerned with the ‘foreign’ versus the ‘indigenous’; ‘otherness’ versus ‘identity’; and ‘modernity’ versus ‘tradition’ components, rather than with concerns about the effectiveness and social acceptability of the bioethical theories and principles themselves in contemporary Africa. In all these oppositional pairs, absolute priority is usually arbitrarily presumed in favour of the latter category in each pair. This tendency suggests a wrong belief that ‘authenticity’ or legitimacy are obvious corollaries of identity; that is, that as long as we can identify with certain principles and ideas (those which are indigenous to us), then such principles and ideas are automatically authentic and, or legitimate while the foreign ones are automatically not.

Now, there are at least two important disclaimers to make at this point: One, there is no doubt that many African contributors to the debate would agree that there are some of the African traditional African values, customs and ways of thinking that are abhorrent in contemporary Africa, so much so that they would be automatically excluded in our thinking about bioethics. But if this is the case, then we cannot be concerned with decolonization as such, nor can we criticize theories and principles for the mere fact of not being indigenous to Africa – more to this later. The second disclaimer is that the above observations should not be construed as denying the importance of the local socio-economic context in discussions and implementation of bioethics. As a recent discussion has shown, the presumptive character of ethical principles and rules implies that cultural and other local nuances can justify departure from, or reinterpretation of and addition to ethical requirements in actual practice [14]. Instead, the remarks are intended as a caution that whereas the search for and preservation of identity is a legitimate consideration in discussions and implementation of bioethics generally, it is neither the only consideration nor, arguably, the most important one. Hence, the priority of the indigenous and traditional values as constituents of identity must be conditional in consideration of other competing values in the cultivation of bioethics in Africa. I will make further remarks on this conditionality later under the *methodological insights* section.

#### Problematic designation for the discourse

A recent discussion cautioned that on-going discussion of bioethics in Africa is guided by a doubtful designation: ‘African bioethics’ [4]. Misgivings about this designation are based on the observation that it sets a defective attitude and points us in a problematic direction characterized by parochialism and prejudice. The unfortunate implication of the current designation as defined above is that Africa needs tailored bioethical principles derived exclusively from an African worldview. To fully appreciate the problem with this designation, its implication can be compared with those of other potential designations. Consider, for example, the one we might frame from a title of one of recent works: ‘*Giving voice to African thought in medical research ethics’* [2]. As opposed to the implications of ‘African bioethics’, this title implies an acknowledgement of Africa’s inescapable engagement with the rest of the world regarding debates and implementation of appropriate global bioethics, amidst ever-growing volume of highly needed North-South collaborative health-related research. It makes the primary aim of bioethics in Africa the untiring projection of Africa’s voice in this engagement. The intellectual attitude it suggests is the same as the title used by Augustine Shutte: *African ethics in a globalizing world*. Under this title the caution is that in an attempt to find a place for African ethics in a globalizing world, ‘there is no avoiding an engagement with European culture and European ethics [...], and if it is a critical engagement, so much the better’ [15]. Both of these candidate designations acknowledge our contemporary geopolitical realities and imply a room for impartiality in the choice of ethical principles for Africa.

In a recent work, an explicitly suggested alternate designation under which discussions of bioethicists in Africa should proceed is ‘Bioethics in Africa’ as opposed to ‘African Bioethics.’ Its authors define it as: ‘[….] an application of bioethical ideas and moral commitments *irrespective of their provenance* in the consideration of moral problems emerging from the effects of globalization on healthcare and human wellbeing in Africa’ [4] (my emphasis). They observe that Moral ideas, theories and principles, whether from Africa, West, or the East are prima facie applicable in so far as they are proportionately and significantly integrative in the resolution of a specific morally problematic situation in Africa. In their view, one of the attractions of ‘bioethics in Africa’ is that it adds to the diversity of bioethical visions and horizons in contemporary times [4]. From these insights it is already clear that a change of designation alone disqualifies some of the current preoccupations with doubtful importance such as concerns with ‘foreign’ versus the ‘indigenous’; ‘otherness’ versus ‘identity’ and ‘modernity’ versus ‘tradition’. Moreover, the title ‘African bioethics’ as defined, directly defeats the spirit of ‘African bioethics’, proclaimed by the Pan-African Bioethics Initiative (PABIN): that is, ‘To advance a global dialogue on Best Practices in Health Research’ [3]. Generally, any designation along the lines of the three candidate titles identified above suggests a better attitude than ‘African bioethics’.

However, it is important to exemplify further how some of the very useful insights into African bioethics are likely to be undermined by the inclusion of some concerns with doubtful importance. Consider, for example, the justification provided for ‘*Giving voice to African thought in medical research ethics’* [2]. On the basis of the above remarks on the designation of the discourse, there is no doubt that amplifying an African voice in discussions of global bioethics is one of the most astute observations about the growth of bioethics in Africa. However, concern remains about the manner in which this crucial aim is justified; that is, the background against which the task of giving voice to African thought in medical research ethics is considered appealing. Instead of insisting on the fact that lack of an African voice in global bioethics means that, for example, such bioethics is missing the positive ethical insights from an African worldview that might enrich bioethics as the author mentions towards the end of that work, the author’s main justification shifts to the consequences of colonial and neocolonial experiences – the dehumanization, exclusion, marginalization, proselytization, indoctrination of Africa and Africans [2]. These are followed by citation of recent evidence that some of the western researchers and some institutions responsible for regulating research such as the World Health Organization (WHO) engage in double standards in implementing existing ethical guidelines in international collaborative medical research [2]. The point to be emphasized about this sort of justification is that even though these are extreme shortcomings in existing bioethics system, they cannot be regarded as justification for rejecting Western principles and theories in themselves or projecting an African voice in global bioethics. In other words, this manner of justification exposes the excellent point about giving voice to African bioethics. This is because it is possible to argue that if this is the justification for ‘African bioethics’, as giving voice to African thought in medical research ethics, we can as well focus on ensuring strict implementation of existing guidelines as they are, since they already forbid the kinds of ills described. That’s why, as I will show below (*Methodological insights* section), on top of focusing on identifying bioethical theories and principles and examining them on their own merit, we need to equally focus on strategies for ensuring rigorous implementation of the resulting bioethics as a solution to the worries reflected in the justification offered for *Giving voice to African thought in medical research ethics*.

#### Negative but hard-to-accept assumption

From a doubtful designation and consequent challenges, some contributions to the discussions of bioethics in Africa reflect, both implicitly and explicitly, a strong negative and yet, in my view, seemingly hard-to-accept assumption. This assumption is that current bioethics and efforts by Western partner institutions to encourage its growth on the continent are all an unholy scheme to create or sustain the Western colonial or neocolonial hegemony, to perpetuate Western dominance and exploitation of the continent [2, 6, 10, 16]. In particular, the suspicion of intentions to exploit would seem fairly credible in a profit-driven global health system, including health-related research and development, as the existing guidelines for research themselves caution [17]. However, the regrettable impact of proceeding on such a negative assumption is that it fosters arbitrary mistrust and bias, all of which negatively impact objectivity as seen, for example, in an uncritical rejection of everything Western while uncritically accommodating everything indigenous and traditional to Africa. It further explains the common cognitive biases prevalent in the discourse particularly ‘in-group/out-group bias’, characterized by the rhetoric of ‘otherizing’, evidenced by use of oppositional terms such ‘they/their’ Versus ‘us/our’ and ‘foreign’ versus ‘local/indigenous’. Even though anger against colonialism, neocolonialism and worst of all, apartheid are good explanations for this attitude, they are not a sound justification for such an attitude in the kind of practical reasoning that ought to guide the growth of bioethics on the continent. This means that effective normative analysis of elements of bioethics in Africa and our chances at influencing global bioethics will depend on our aptitude and willingness to identify and guard against such potential cognitive distortions that undermine and sabotage impartial critical analysis. This task needs to begin with a critical examination of the existing negative assumption about the aim of the current bioethics movement in Africa and the rest of the non-western world.

Prima facie, it makes good sense that given the history of colonization through evangelization to ‘save the souls’ of, and ‘civilize’ Africans and the contemptible consequences that resulted, the persisting traces of racial mistrusts and biases, as well as the real threat of exploitation, Africans should never trust the West, its agents as well as Western ideas. The evidence of double standards in adhering to or implementing existing ethical guidelines and principles by the west cited earlier, not to mention recent allegations of disguised sterilization of Kenyan women by the United Nations International Children’s Emergency Fund (UNICEF) [18], all seem to further strengthen the mistrust. This being the case, proceeding on this negative assumption becomes a matter of caution. Therefore, one might contend that even if this mistrust could be an error, it is better to err on the side of caution.

However, the purported justification of mistrust ignores the very atmosphere in which existing bioethical principles arose; just to cite two cases that moreover reek of racism – the Tuskegee Syphilis study [19] and the Nazi scientists’ experiments on the Jews and Romany peoples [20, 21]. These and many other cases are cited as drivers of modern bioethics, particularly of guidelines for conducting health-related research involving human participants, much so the vulnerable ones. In other words, contemporary bioethics is, in principle, to say the least, a condemnation of such historical practices and at the same time an evolving effort to always caution and guard against similar ones now and in future. In other words, it is the same Western world that first articulated and implemented ethical rules to guide and evaluate the moral acceptability of subsequent studies with the aim of guarding against dehumanization, exploitation and all forms of injustice; disrespect of individuals’ rights to self-determination; minimizing potential harms to participants and guaranteeing benefits to vulnerable populations - including those in Africa. It is for this reason that charges of double standards based on evidence of some Western researchers’ misconduct as justification for rejection of Western bioethics fail. This purported justification fails because it does not distinguish between the merits of principles *qua* principles on the one the hand, and specific institutional failures to implement such principles to the letter, on the other. For this reason, it is surprising that instead of insisting on the strict implementation of these safeguards, some African bioethicists, as quoted elsewhere, charge that the West’s insistence on strict implementation of these very ethical principles and procedures in developing countries ‘reeks of ethical imperialism’ [22]. In summary, a combination of the historical drivers of existing bioethics combined with a need to distinguish the merits of principles from institutional failure to implement them to the letter permits us to infer that the negative assumption underlying some contributions to discussions of bioethics in Africa along with the mistrust it begets lack plausibility.

#### Indeterminate problem and aims

Another critical observation made about bioethics in Africa is a lack of direction for its growth [1]. Generally, the clarity of direction of any discourse largely depends on the degree of precision with which its problem, aim(s) and foundational question(s) are defined. However, a number of contributions to discussions of bioethics in Africa are of such a nature that once carefully studied, one will most likely wonder what exactly the issue is. So far it is not clear whether the rejection of existing so-called Western bioethics is a rejection of principles *qua* principles and practices based on them, or simply a rejection of the authors of these principles by virtue of the attributes of those authors. I have already mentioned that if the answer to this question is affirmative, this is a flawed attitude. In the first place, random rejection of ‘Western bioethics’ or ‘principlism’ seems unconscious because certainly Africans are averse to harms and exploitation and have concepts of justice, benefit and altruism. Hence, it is not clear whether by rejecting Western bioethics we actually mean to reject the Western principle-based bioethics as a whole, or just one principle – respect for individual autonomy. Even on autonomy it is not clear whether we intend to say the all health-related decisions and practices based on respect for individual autonomy or the philosophy of individual liberalism generally, are alien and unwelcome in Africa or, if we intend to say that *all things being equal* decisions and practices based on communitarian values take precedence over those based on individual liberalism. Further, as indicated earlier, sometimes the aim of ‘African bioethics’ is portrayed as an attempt to influence or reform international (global) bioethics using worthwhile traditional African moral insights [15]; at other times ‘to build customized bioethics system for Africa’ [15]. In other instances, the goal seems to be ‘to challenge the double-standards of the West’ [15]. Another aim, which deserves attention, is to discover and articulate vital ethical principles and theories from an African worldview; principles that can be used to effectively address contemporary African health needs and challenges’ [1, 4, 16]. This direction has been successfully demonstrated by an articulation of the ‘Human Life Invaluableness’ as an emerging African bioethical principle [7] as well as Thaddeus Metz’s attempt at articulating an African theory of bioethics [8].

However, I should clarify that from the foregoing remarks about the seemingly many aims reflected in discussions of bioethics in Africa, the intended point is not that it is inappropriate for a discourse to address objectives or questions as many as those identified in discussions of bioethics in Africa. However, in the case of views on bioethics in Africa, the problem is that some of the aims are either incompatible with each other, while others are outrightly inappropriate. For example, the pursuit of ‘identity,’ ‘tradition’ and the ‘indigenous’ that arbitrarily presumes their absolute priority over their opposites, will, in many cases, conflict with the requirement of an impartial search for effective ethical principles for contemporary Africa irrespective of the origin of such principles. On the other hand, concerns and arguments of ‘who is to say’ (*argumentum* ad hominem) are automatically inappropriate in the sort of practical reasoning to guide bioethics discussions, including on the African continent they divert our attention from an examination of principles *qua* principles (and, or theories) to immaterial considerations such as the origin of theories and principles and attributes of their authors.

#### The rhetoric of ‘Africanity’

The general issue with rhetoric, whether used consciously or inadvertently, is its hard-to-resist negative impact on the exercise of rationality and objectivity. This impact, however, is difficult to detect in most cases [23, 24]. So far, apart from the language of ‘otherizing’ ‘demonizing’ and ‘in-group/out-group biases’ as forms of rhetoric, some scholarly works on African bioethics tend to use the rhetoric of ‘Africanity’ or ‘Africanness’ (or words with equivalent emotional appeal) to persuade their audiences. In the search for identity, and, or ‘authenticity’ of bioethics in Africa, some contributors argue that existing bioethics lacks ‘Africanity’ or ‘Africanness’. It should not be difficult to see the strong emotional appeal of a combination of this concept with the rest of the rhetorical language so far identified. But what exactly do we mean by ‘Africanity’? The likely definition could be, in my view, that ‘Africanity’ is a set of beliefs, values, and practices representative of African indigenous and traditional ways of living that distinguish Africa from the rest of the world *in relevant and fundamental ways*. However, given the size and cultural and religious diversity of the African continent, it is not easy to specify what exactly these elements could be. For example, we (Africans) happily identify ourselves as deeply religious. But given the religious diversity of contemporary Africa – Islam, Christianity, and African Traditional Religions among others – sometimes with contradicting beliefs and practices, which religion should be used as a marker of ‘Africanity’? Even within the various Christian sects alone, there are fundamental disagreements relevant to health, such as the use of contraception. And further, in the case of traditional norms, values and practices, will it be the Cameroon’s Nso’ traditional beliefs or those of the Banyankole ethnic group of Western Uganda, or the Yoruba traditions from Nigeria? Even within the same country like Uganda (I am Ugandan), for example, there is deeply divided opinion on the moral acceptability of euthanasia among different ethnic groups; differences not based on their Christian or Islamic or other foreign religious sentiments, but on their indigenous and traditional beliefs. And what about the traditional health and medical-related beliefs and practices which are regarded by Christians as satanic; for example, some traditional bone-setting instances in which traditional healers effectively do their job without ever coming into physical contact with, or ever seeing their patients? Interestingly, rhetoric similar to that of ‘Africanity’ was sadly noted in human rights debates in Asia in which regimes with dubious human rights records appeal to ‘Asian values’ in their attempt to wrongly portray human rights and Asian values as being incompatible [25]. In Japan, related rhetoric under the title of ‘*mutual dependency’* as the defining morality of Japanese society was reported to be used to illegitimately suppress public opinions on the controversy of brain death and attitudes towards euthanasia, consequently stifling the possibility of critical interrogation of related issues [26].

Most likely, the charge against the above observation will appeal to Ubuntu philosophy as a universal marker of ‘Africanity’ and which distinguishes Africa from the rest of the world in a *relevant and fundamental way*. However, such a response will be begging the question. Generally, the gist of Ubuntu philosophy is the ethos of being each other’s keepers and its related communitarian philosophy, yet these tenets are not limited to the African continent. For example, many major world religious traditions – Hinduism, Christianity, Islam, Buddhism, Confucianism among others – in one way or another sought (and still do) to redirect their adherents from self-centeredness towards caring about the needs of others [27]. In the case of communitarian philosophy it is not true that communitarian sympathies are limited to Africa or were borrowed from Africa wherever they exist, including the United States of America and Europe. Even though there could be evidence that communitarian values are still much stronger in Africa than in Western societies, it is not clear whether, for example, they are stronger than they are in China. But that said, we further need to bear in mind the liberal human rights tradition that is already deeply entrenched in most African public institutions and deeply internalized by Africans. In other words, what may be asserted as ‘Africanity’ in a sense of its ability to distinguish Africa from the rest of the world in a *fundamental and relevant sense* to justify a blanket rejection of the whole of the Western bioethics in pursuit of ‘Africanity’ is not clear. Subsequently, since there loom deeper disagreements on the fundamental elements of African bioethics among Africans themselves, charges of ‘ethical imperialism’ between different cultural and religious groups within the continent, even within countries, await ‘African bioethics’ as a set of customized bioethical principles based on indigenous and traditional beliefs and values. So, appeal to ‘Africanity’ as a justification for a rejection of existing bioethics and thereby coining customized African bioethics seems for the most part to be more of rhetoric, the impact of which we ought to be critically conscious of in our discussions of bioethics in Africa.

### Methodological insights for bioethics in Africa

The above discussion was intended to confirm the claim about the lack of clear direction in the growth of bioethics in Africa. It is on the basis of this confirmation that the recommendation of ‘the need for a robust methodological inquiry in bioethics in Africa’ [4] becomes compelling. Since bioethics in Africa needs to be both effective and socially acceptable, among other potential aims, a sound method for cultivating bioethics in Africa ought to balance at least five aims: i) Cautious pursuit of identity ii) identifying ethical principles effectiveness in addressing the health needs and challenges of *contemporary* Africa in a morally sound manner; iii) ensuring that the principles chosen and practices based on them are socially acceptable to *contemporary* Africans and iv) an attempt to reform and enrich global bioethics theory and practice using positive moral insights from an African worldview and, over and above all, V) workout strategies for strict implementation of resulting bioethics.

#### Cautious pursuit of identity

For easy implementation of bioethics in Africa and in consideration of the requirement to respect communities, a search for cultural identity with the intention of improving the social acceptability of bioethics in Africa becomes a legitimate aim. However, as hinted earlier, caution is warranted: identity *as such* ought not to be the only, neither, arguably, the most important goal of the discourse. Given that context is crucial in the choice, interpretation and implementation of ethical principles in local contexts [14], discussions of bioethics in Africa can legitimately proceed on a *conditional presumption* of priority of an African worldview. However, a presumption favoring the priority of an African worldview does not mean that all ethical theories, principles, beliefs and values derived from an African worldview *must always* trump Western or other non-African moral categories, as widely implied in current discussions. Instead, the condition is simply that *all things being equal*, in discussions, teaching and implementation of bioethics in Africa, worthwhile moral theories, principles and values that reflect an African worldview should take precedence over non-African values. The proviso *all things being equal* must be taken seriously for two reasons. One, it is caution against uncritical acceptance of all traditional and, or indigenous moral objects. Secondly, it is intended to guard against credulous denunciation of all foreign moral categories for the simple fact of being foreign. In a sense, the proviso further implies that there is nothing inherently wrong with a people protecting their heritage and identity as such and at the same time it emphasizes the importance of objectivity and impartiality in discussions of bioethics. Generally, the proviso implies that there are potentially other important considerations that can legitimately override concerns of identity and preservation of some aspects of a society’s heritage.

#### Engage with principles qua principles

Arguments about ‘who is to say’ or ‘*argumentum ad hominem’* are usually suspect in moral theorizing and practical reasoning generally. Candidate bioethical principles ought to be examined on their own merit on the basis of pre-determined criteria. Therefore, we need to be primarily concerned with identifying and evaluating competing bioethical principles in light of Africa’s contemporary needs and situatedness. In short, the process of engaging with principles *qua* principles can imitate the following procedure:

1. Engage in an impartial analysis of the health needs and related moral challenges in contemporary Africa. This analysis must consider the *contemporary* socio-economic as well as geopolitical environment within which African health systems operate. For example, the severe scarcity of health resources on the continent will make it mandatory for Africans to search for ethical principles to deal with existing gross inequities in current allocations of available resources [28]. The consideration of resources is also important in decisions regarding morally acceptable compromises to make about, say, ‘Standard of Care’; the moral acceptability of placebo-controlled clinical trials in Africa [29, 30]; decisions regarding the scope and obligations for ancillary care and incidental findings among other bioethical controversies that have resource implications in health research. Further, the discussions relating to health resources would be especially crucial if a proposed study is urgently needed in a country without any care for the target condition and yet the provision of a high standard of care or broad ancillary care as requirements for that study are impossible due scarcity of resources, as can be seen in debate about the formerly controversial AZT clinical trials in developing countries [15].
2. In the analysis of health challenges facing contemporary Africa, we could list all candidate bioethical theories and principles that can potentially respond effectively to the identified needs and moral challenges – whether in*,* inter alia, health research and development (R&D); medical/clinical practice; public health and priority setting in health resource allocation. These principles should be listed irrespective of who articulated them first.
3. Thirdly, there ought to be criteria against which each of the competing principles is to be examined. These criteria must, inter alia, involve items relating to potential effectiveness and social acceptability of each of the contending principles in Africa’s contemporary realities.
4. The fourth step would then be an actual impartial examination of each of the competing ethical principles against the set criteria.

#### Complementing and implementing existing bioethics

One of the major motivations for the trend towards ‘African bioethics’ is the real threat posed by weak regulation and related consequences – exploitation, dehumanization, injustice, double-standards, name it. However, the feared ills cannot be avoided by rejecting Western bioethics as such, but perhaps by its strict implementation. Of course we need to acknowledge the possibility that existing (Western) bioethics will be insufficient or inappropriate in some cases. Hence, on top of complementing and reforming existing bioethics using worthwhile African moral insights where necessary, there is need to work out a strategy-cum-mechanism for strict implementation of resulting bioethics, especially whenever such implementation is taking place in Africa.

#### Emphasis of the contemporary

The emphasis on contemporary circumstances is critical for at least two reasons. One, if the principles are to be effective, they need to acknowledge the changes taking place in African health systems, such as increasing reliance on modern medicine as opposed to traditional healing; the growing type and volume of health-related research on the continent; not forgetting several controversial issues raised by emerging biotechnologies. Secondly, the importance of the contemporary is already partly demonstrated by, for example, a study of the mostly controversial principle of ‘respect for individual autonomy’, especially in obtaining informed consent from participants in health-related research in rural Ghana [31]. This study investigated attitudes towards the process of obtaining informed consent, in which one of the questions investigated attitudes towards ‘Community’ versus ‘Individual’ consent. Whereas some contributors to African bioethics, particularly in research ethics, claim that Africa’s communitarian values make ‘individual consent’ an alien concept and practice as opposed to community consent, most participants in this study insisted that community consent by, say, a village/community chief or clan leader (gatekeeper) though very important, cannot replace individual consent. This attitude was shared by most of the chiefs interviewed. The fact that this study was conducted in a rural setting where 90% of the participants had not attained any sort of formal education disqualifies the potential charge that such an attitude is a result of Western manipulative influence. Other studies will most likely show, as one has already done, that African women are no longer willing, if they ever were, to be treated as less competent than their husbands in giving informed consent to a point of being required or even strongly encouraged to consult their husbands in the process of making such decisions, while the same demand does not equally apply to men [32].

#### Cultivate critical thinking skills and habits

Bioethics is moral reasoning applied to life sciences, biotechnologies, health and medical care and all resulting practices. Being applied moral reasoning, discussions in bioethics require certain cognitive skills which can be deliberately cultivated. Hence there is need to train ourselves in, inter alia, how to immediately detect and steer clear of our own cognitive limitations (especially psychological biases) and those of others; how to identify and articulate issues and what it takes to prove a point on an issue; that is, what evidence counts. Uncertainty about what the issue(s) is/are, makes it impossible to be clear on what one’s argument should be and what kind of evidence is needed to demonstrate a point or respond to it. Against this backdrop, it is hoped that the discussion above demonstrates that the importance of teaching critical thinking to bioethicists can never be overstated.

## Conclusion

Unlike the West and some other parts of the world, the growth of bioethics in Africa is at a nascent and critical stage. It is a critical stage because documented knowledge and practices of bioethics are still relatively new in many parts of Africa, seeking to grow wider and deeper. In other words, bioethics in much of Africa is still at a foundational stage and this calls for efforts to establish a strong foundation for its growth on the continent. The discussion above has attempted to demonstrate how weak this foundation still is and how a lack of such a firm and clear foundation and direction threatens to distort the essence and development of bioethics on the continent. For this reason, in order to forestall the possibility of distorted development and misrepresentations of bioethics in Africa based solely and narrowly on a mere search for identity, the implementation and teaching of bioethics in Africa needs to be critically assessed and guided in a clear and consciously desirable direction. Bioethics in Africa will develop faster and better if we start to worry less about who first articulated which bioethical principles and, instead, start to focus more on how applicable such principles are in solving contemporary Africa’s many health challenges and devising strategies for ensuring strict implementation of resulting bioethics. The successful accomplishment of this task will take Africa a long way towards identifying, teaching and implementing pragmatic ethical principles and amplifying Africa’s voice in global discussions of bioethics.

## Abbreviations

HIV/AIDS: Human Immunodeficiency Virus/ Acquired Immunodeficiency Syndrome
PABIN: Pan-African Bioethics Initiative
UNICEF: United Nations International Children’s Emergency Fund
WHO: World Health Organization
